# Changes in body composition and handgrip strength during dapagliflozin administration in patients with chronic kidney disease

**DOI:** 10.1093/ckj/sfaf075

**Published:** 2025-03-12

**Authors:** Takahiro Yajima, Kaoru Noda, Kumiko Yajima

**Affiliations:** Department of Nephrology, Matsunami General Hospital, Gifu, Japan; Department of Internal Medicine, Matsunami General Hospital, Gifu, Japan; Department of Internal Medicine, Matsunami General Hospital, Gifu, Japan

**Keywords:** dapagliflozin, extracellular water-to-total body water ratio, handgrip strength, sarcopenia, skeletal muscle index

## Abstract

**Background:**

Dapagliflozin improves renal endpoints; however, concerns exist regarding safety in patients with chronic kidney disease. We examined the effects of dapagliflozin on body composition, handgrip strength, and sarcopenia.

**Methods:**

This prospective observational study included 55 patients with chronic kidney disease (type 2 diabetes, *n *= 27) treated with dapagliflozin 10 mg/day for 24 weeks. Handgrip strength, bio-impedance analysis-estimated skeletal muscle index, and extracellular water-to-total body water ratio were measured at baseline, 12 weeks, and 24 weeks after dapagliflozin administration. Sarcopenia was diagnosed as reduced handgrip strength (women: <18 kg; men: <28 kg) and decreased skeletal muscle index (women: <5.7 kg/m^2^; men: <7.0 kg/m^2^).

**Results:**

During dapagliflozin therapy, skeletal muscle index significantly decreased from 7.51 ± 1.36 kg/m^2^ at baseline to 7.40 ± 1.28 kg/m^2^ at 12 weeks (*P < *0.0001) and 7.32 ± 1.35 kg/m^2^ at 24 weeks (*P < *0.0001). The extracellular water-to-total body water ratio decreased from 0.391 ± 0.012 at baseline to 0.390 ± 0.011 at 12 weeks (*P = *0.17) and 0.389 ± 0.010 at 24 weeks (*P = *0.002). Conversely, handgrip strength was unchanged from 32.9 ± 12.2 kg at baseline to 34.0 ± 12.1 kg at 12 weeks (*P = *0.022) and 33.9 ± 12.4 kg at 24 weeks (*P = *0.14). Sarcopenia prevalence did not change during dapagliflozin treatment [10.9% (*n *= 6) at baseline, 14.5% (*n *= 8) at 12 weeks, 10.9% (*n *= 6) at 24 weeks; *P = *0.45.

**Conclusions:**

During the 24-week dapagliflozin treatment, there was a decrease in skeletal muscle index and extracellular water-to-total body water ratio with unchanged handgrip strength in patients with chronic kidney disease. Sarcopenia prevalence remained constant. Therefore, regarding sarcopenia, short-term dapagliflozin administration might be safe. However, further long-term studies are required to determine the safety of dapagliflozin in these patients.

## Graphical Abstract

**Graphical Abstract:**
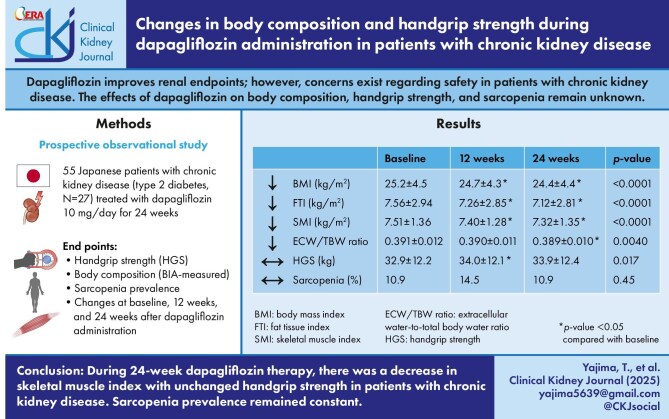

KEY LEARNING POINTS **What was known:**

- Chronic kidney disease is a critical health concern, particularly in patients with type 2 diabetes.
- Sodium–glucose cotransporter 2 (SGLT2) inhibitors reduce body weight, fat mass, and even skeletal muscle mass in patients with type 2 diabetes. Though dapagliflozin, a SGLT2 inhibitor, improves renal endpoints, there might be a safety concern regarding sarcopenia in patients with chronic kidney disease.

**This study adds:**

- During the 24-week dapagliflozin therapy there was a decrease in the skeletal muscle index and extracellular water-to-total body water ratio, with unchanged handgrip strength.
- Sarcopenia prevalence remains constant in patients with chronic kidney disease after dapagliflozin treatment.

**Potential impact:**

- Short-term dapagliflozin administration might be safe in patients with chronic kidney disease. However, further studies with long-term follow-up are required to determine the safety of dapagliflozin in these patients.

## INTRODUCTION

Sodium–glucose cotransporter 2 (SGLT2) inhibitors, widely used to treat type 2 diabetes, can lower blood pressure, blood glucose, and uric acid and protect the heart and kidney [1, 2]. SGLT2 inhibitors reduce body weight, fat mass, and skeletal muscle mass in patients with type 2 diabetes [3, 4]. It is hypothesized that SGLT2 inhibitor-induced urinary glucose loss reduces insulin levels and restricts the uptake of glucose and amino acids into the muscle, and elevates glucagon levels and enhances proteolysis in skeletal muscle [5, 6]. This pathophysiology might lead to sarcopenia, a concomitant reduction of muscle strength and mass volume [7, 8]. However, handgrip strength (HGS) data remain largely unreported, and therefore the relationship between SGLT2 inhibitors and sarcopenia in patients with type 2 diabetes mellitus remains unclear. A DAPA-CKD trial reported that dapagliflozin delays chronic kidney disease (CKD) progression [9], expanding the clinical indications of dapagliflozin to patients with CKD (with or without diabetes). However, the CKD population is at high risk of sarcopenia [10, 11]; a negative protein balance due to accelerated protein catabolism from CKD itself, with chronic inflammation and decreased protein and energy intake, is related to sarcopenia in these patients [10]. However, the effect of dapagliflozin on sarcopenia remains unclear in CKD patients (with or without type 2 diabetes).

We hypothesized that dapagliflozin might exacerbate sarcopenia in this population and examined the impact of dapagliflozin on HGS, bioimpedance analysis-estimated body composition including fat mass and muscle mass, and sarcopenia prevalence in patients with CKD.

## MATERIALS AND METHODS

### Study design and participants

We conducted a single-center, open-label, single-arm, prospective, 24-week observational study at Matsunami General Hospital between January 2022 and December 2023. We enrolled CKD patients with an estimated glomerular filtration rate (eGFR) of 15–60 mL/min/1.73 m^2^ and/or a urinary protein-to-creatinine ratio (UPCR) >0.15 g/gCr [or a urinary albumin-to-creatinine ratio (UACR) >30 mg/gCr] for ≥3 months. The patients received regular nutritional consultations and were treated with the same medication regimen for ≥3 months. We excluded patients with type 1 diabetes mellitus; a history of diabetic ketoacidosis; genital infection or urinary tract infection; age <20 and >80 years; treatment of malignancy; inability to undergo body composition evaluation using bio-impedance analysis due to pacemaker implantation or inability to remain standing during the examination; and previous administration of SGLT2 inhibitors. The study adhered to the Declaration of Helsinki principles and was approved by the Ethics Committee of Matsunami Hospital (approval no. 512). Written informed consent was obtained from all patients.

### Study protocol

Patients received dapagliflozin (10 mg/day) for 24 weeks. Blood and urine samples were collected, and body composition and HGS were measured before and 12 and 24 weeks after dapagliflozin administration. Diet therapy [energy intake 30 kcal/ideal body weight (kg)/day] was provided by fixed experienced dietitians at baseline, 12, and 24 weeks. Based on the dietary recommendations for chronic kidney disease 2014 [12], a recommended protein intake was set by CKD staging: CKD stage G3a: 0.8–1.0 g/ideal body weight (kg)/day; G3b, G4, and G5: 0.6–0.8 g/ideal body weight (kg)/day. Accounting for the risk of sarcopenia, the upper limit of recommended protein intake was applied in the present study: CKD stage G3a: 1.0 g/ideal body weight (kg)/day; G3b, G4, and G5: 0.8 g/ideal body weight (kg)/day. For exercise therapy, walking for >30 min at least four times weekly was recommended. As for other lifestyle factors, quitting smoking and alcohol consumption, and drinking enough fluid intake (not specified but >500–1000 mL more water) to avoid dehydration were recommended.

### Body composition measurements

For assessing body composition, a multifrequency body composition analyzer (InBody 770; InBody Japan Inc., Tokyo, Japan) was used. Fat mass, skeletal muscle mass, and the ratio of extracellular water to total body water (ECW/TBW ratio) were measured. The fat tissue index (FTI) and skeletal muscle index (SMI) were calculated as follows: FTI, fat mass/height squared (kg/m^2^); SMI, skeletal muscle mass/height squared (kg/m^2^).

### HGS measurements

HGS was measured twice for each hand using a grip dynamometer (Hand Dynamometer, Smedley type, AS ONE Corporation, Osaka, Japan). The average of maximum right- and left-hand HGS values was used as HGS value.

### Sarcopenia diagnosis

Sarcopenia was diagnosed based on the Asian Working Group for Sarcopenia 2019 (AWGS 2019) consensus [8], defined as decreased HGS (women: <18 kg; men: <28 kg) and reduced SMI (women: <5.7 kg/m^2^; men: <7.0 kg/m^2^).

### Study outcomes

Primary outcomes included longitudinal changes in body mass index (BMI), FTI, SMI, ECW/TBW ratio, HGS, and the prevalence of sarcopenia at baseline, 12, and 24 weeks while taking dapagliflozin 10 mg/day. Comparisons were made between patients with and without type 2 diabetes. Secondary outcomes included changes in eGFR, UPCR, UACR, HbA1c, and glycated albumin (GA) at baseline, 12, and 24 weeks. Additionally, side effects were monitored throughout the study.

### Statistical analyses

Pre-specific sample size calculations were not conducted because of the nature of this single-arm study. We performed *post*  *hoc* analysis to evaluate whether the sample size had sufficient statistical power to detect differences before and after dapagliflozin therapy to change SMI and HGS. The *post*  *hoc* statistical power was 17.5% for SMI (mean difference: 0.19 kg/m^2^; SD: 1.35 kg/m^2^) and 8.7% for HGS (mean difference: 1.0 kg; SD: 12.2 kg) using a two-sided significance level of *P* < 0.05 for differences in changes at baseline and 24 weeks.

A skewed distributed variable is expressed as the median (interquartile range), and a normally distributed variable is expressed as mean ± standard deviation. Baseline characteristics of patients with and without type 2 diabetes were compared using the Mann–Whitney *U*-test or Student's *t*-test for continuous data and Fisher's exact test or Pearson's χ^2^ test for categorical data. One-way repeated measures ANOVA was applied to normally distributed data; Friedman's test was applied to skewed distributed data. When the *P*-value was <0.05, Bonferroni correction was performed for multiple comparisons. Two-way repeated measures ANOVA was applied to examine the interaction of changes between diabetes and non-diabetes groups at baseline, 12, and 24 weeks. Additionally, subgroup analyses stratified by age (<65 vs ≥65 years) or baseline prescription of renin-angiotensin system (RAS) inhibitors and statins were conducted. A *P*-value of <0.15 in the interaction test was considered clinically significant. Cochran's Q test was applied to assess differences in the proportion of patients with sarcopenia at baseline, 12, and 24 weeks in all patients and those with and without type 2 diabetes. Pearson's correlation coefficient was applied to examine correlations among percentage changes during the 24-week BMI and body composition. All statistical analyses were performed using SPSS version 28 (IBM Corp., Armonk, NY, USA). Statistical significance was set at *P < *0.05.

## RESULTS

### Clinical characteristics

This study enrolled 60 CKD patients; however, five patients who discontinued taking dapagliflozin were excluded due to poor adherence (*n *= 2); dropping out from the outpatient clinic (*n *= 2); and admission due to operation for an incidentally discovered iliac aneurysm (*n *= 1). The remaining 55 patients who continued dapagliflozin treatment were included in the data analyses.

Table 1 lists the characteristics of participants. Men constituted 74.5%, with a mean age of 62.3 years, and approximately half (*n *= 27, 49.1%) of them had type 2 diabetes. Patients with type 2 diabetes received dietary and exercise therapy (*n *= 5, 18.5%), dipeptidyl peptidase-4 inhibitor (DPP4i) (*n *= 12, 44.4%), DPP4i, and metformin (*n *= 3, 11.1%), DPP4i and glinide (*n *= 1, 3.7%), insulin (*n *= 2, 7.4%), insulin and DPP4i (*n *= 3, 11.1%), and insulin and glucagon-like peptide-1 receptor agonist (GLP-1RA) (*n *= 1, 3.7%) at enrollment. Basal kidney diseases other than diabetic kidney disease were as follows: chronic glomerulonephritis, *n *= 15 (53.6%); nephrosclerosis, *n *= 11 (39.3%); drug-induced, *n *= 2 (7.1%). Patients with diabetes tended to be older, frequently took diuretics, and had lower cholesterol levels than those without diabetes. Additionally, blood glucose, HbA1c, GA, UPCR, and UACR were significantly higher in patients with type 2 diabetes than those without. In contrast, the BMI, FTI, SMI, and HGS were comparable between the groups. The baseline ECW/TBW ratio was higher in patients with than in those without type 2 diabetes. The baseline prevalence of sarcopenia tended to be higher in patients with diabetes (18.5%, *n *= 5) than in those without diabetes (3.6%, *n *= 1) (*P = *0.075).

**Table 1:** Baseline characteristics of the study participants.

	All patients	Patients with non-diabetes	Patients with diabetes	
	(*n *= 55)	(*n *= 28)	(*n *= 27)	*P*-value
Men (%)	74.5	67.9	81.5	0.24
Age (years)	62.3 ± 11.8	59.4 ± 11.1	65.4 ± 11.9	0.055
Alcohol (%)	34.5	25.0	44.4	0.13
Smoking (%)	23.6	17.9	29.6	0.30
Hypertension (%)	83.6	78.6	88.9	0.30
Hyperlipidemia (%)	61.8	57.1	66.7	0.47
Hyperuricemia (%)	25.5	17.9	33.3	0.19
Cardiovascular disease history (%)	12.7	7.1	18.5	0.20
Medications				
RAS inhibitors (%)	60.0	53.6	66.7	0.32
Diuretics (%)	16.4	7.1	25.9	0.054
Statin (%)	50.9	46.4	55.6	0.50
Body weight (kg)	69.1 ± 16.7	69.0 ± 19.8	69.3 ± 13.1	0.94
Body mass index (kg/m^2^)	25.2 ± 4.5	25.2 ± 5.2	25.3 ± 3.9	0.94
Albumin (g/dL)	4.1 ± 0.3	4.1 ± 0.3	4.0 ± 0.4	0.13
Total cholesterol (mg/dL)	203 ± 34	212 ± 29	194 ± 37	0.054
Hemoglobin (g/dL)	13.9 ± 1.9	14.3 ± 1.5	13.5 ± 2.1	0.10
Creatinine (mg/dL)	1.41 ± 0.55	1.36 ± 0.53	1.46 ± 0.57	0.51
eGFR (mL/min/1.73 m^2^)	43.9 ± 17.1	45.3 ± 17.7	42.6 ± 16.7	0.56
Blood glucose (mg/dL)	141 ± 50	115 ± 30	168 ± 52	<0.0001
HbA1c (%)	6.3 ± 1.0	5.7 ± 0.4	7.0 ± 1.0	<0.0001
Glycated albumin (%)	15.8 ± 4.2	13.5 ± 1.7	18.2 ± 4.6	<0.0001
Brain natriuretic peptide (pg/mL)	27 (10–77)	23 (10–75)	27 (11–125)	0.53
Urinary protein:creatinine ratio (g/gCr)	0.7 (0.3–1.7)	0.6 (0.2–1.4)	0.8 (0.4–4.5)	0.019
Urinary albumin:creatinine ratio (mg/gCr)	540 (150–1266)	371 (63–982)	574 (209–2865)	0.022
Fat mass (kg)	20.5 ± 8.5	19.9 ± 9.6	21.1 ± 7.3	0.60
Fat tissue index (kg/m^2^)	7.56 ± 2.94	7.29 ± 3.12	7.84 ± 2.78	0.50
Skeletal muscle mass (kg)	26.6 ± 6.7	26.9 ± 7.8	26.3 ± 5.5	0.73
Skeletal mass index (kg/m^2^)	7.51 ± 1.36	7.49 ± 1.59	7.54 ± 1.10	0.89
Intracellular water (kg)	21.9 ± 5.2	22.2 ± 6.0	21.6 ± 4.2	0.67
Extracellular water (kg)	14.0 ± 3.1	14.0 ± 3.7	14.0 ± 2.6	0.99
Total body water (kg)	35.9 ± 8.3	36.2 ± 9.7	35.6 ± 6.7	0.79
Extracellular water:total body water ratio	0.391 ± 0.012	0.387 ± 0.009	0.395 ± 0.014	0.022
Handgrip strength (kg)	32.9 ± 12.2	35.4 ± 12.2	30.3 ± 11.8	0.12
Prevalence of sarcopenia (%)	10.9	3.6	18.5	0.075

### Follow-up study

Patients who received RAS inhibitors and statins at baseline continued to use these medications during the follow-up period. Conversely, a diuretic was prescribed to nine patients at baseline; six patients continued to use it and two patients used it with a decreased dose during follow-up periods, and one patient quit it with the initiation of dapagliflozin administration. Additionally, two patients began to use diuretics because of the exacerbation of edema. However, in this study, only one male patient with diabetes continued to receive GLP-1RA.

### Changes in body composition and handgrip strength during 24-week dapagliflozin therapy in all patients

During the 24-week dapagliflozin therapy, BMI, FTI, SMI, ECW/TBW ratio, and HGS significantly changed in all patients (Table 2). BMI significantly decreased from 25.2 ± 4.5 kg/m^2^ at baseline to 24.7 ± 4.3 kg/m^2^ at 12 weeks (*P < *0.0001) and 24.4 ± 4.4 kg/m^2^ at 24 weeks (*P < *0.0001). FTI also significantly decreased from 7.56 ± 2.94 kg/m^2^ at baseline to 7.26 ± 2.85 kg/m^2^ at 12 weeks (*P < *0.0001) and 7.12 ± 2.81 kg/m^2^ at 24 weeks (*P < *0.0001). The SMI significantly decreased from 7.51 ± 1.36 kg/m^2^ at baseline to 7.40 ± 1.28 kg/m^2^ at 12 weeks (*P < *0.0001) and 7.32 ± 1.35 kg/m^2^ at 24 weeks (*P < *0.0001). Additionally, the ECW/TBW ratio decreased from 0.391 ± 0.012 at baseline to 0.390 ± 0.011 at 12 weeks (*P = *0.17) and 0.389 ± 0.010 at 24 weeks (*P = *0.002). Conversely, HGS significantly increased from 32.9 ± 12.2 at baseline to 34.0 ± 12.1 at 12 weeks (*P = *0.022) and tended to increase to 33.9 ± 12.4 at 24 weeks (*P = *0.14).

**Table 2:** Changes in BMI, bio-impedance analysis-estimated body composition, and HGS after administration of dapagliflozin in patients with CKD.

Variables	Baseline	At 12 weeks	At 24 weeks	*P*-value	*P*-value for interaction
Overall patients					
BMI	25.2 ± 4.5	24.7 ± 4.3	24.4 ± 4.4	<0.0001	
FTI	7.56 ± 2.94	7.26 ± 2.85	7.12 ± 2.81	<0.0001	
SMI	7.51 ± 1.36	7.40 ± 1.28	7.32 ± 1.35	<0.0001	
ECW/TBW ratio	0.391 ± 0.012	0.390 ± 0.011	0.389 ± 0.010	0.0040	
HGS	32.9 ± 12.2	34.0 ± 12.1	33.9 ± 12.4	0.017	
Subgroup analyses					
BMI					0.40
Non-diabetes (*n* = 28)	25.2 ± 5.2	24.7 ± 4.8	24.5 ± 5.0	<0.0001	
Diabetes (*n* = 27)	25.3 ± 3.9	24.7 ± 3.8	24.3 ± 3.8	<0.0001	
FTI					0.99
Non-diabetes	7.29 ± 3.12	6.99 ± 2.97	6.85 ± 3.01	0.001	
Diabetes	7.82 ± 2.79	7.52 ± 2.76	7.39 ± 2.61	0.001	
SMI					0.58
Non-diabetes	7.49 ± 1.59	7.40 ± 1.54	7.34 ± 1.57	<0.0001	
Diabetes	7.54 ± 1.10	7.39 ± 0.96	7.31 ± 1.10	0.016	
ECW/TBW ratio					0.070
Non-diabetes	0.387 ± 0.009	0.387 ± 0.008	0.386 ± 0.009	0.37	
Diabetes	0.395 ± 0.014	0.393 ± 0.014	0.391 ± 0.012	0.011	
HGS					0.12
Non-diabetes	35.4 ± 12.2	35.8 ± 12.2	35.6 ± 11.8	0.58	
Diabetes	30.3 ± 11.8	32.0 ± 12.1	32.2 ± 12.9	0.027	
BMI					0.41
Age <65 years (*n = 26*)	26.9 ± 5.1	26.3 ± 4.8	26.1 ± 5.0	0.0003	
Age ≥65 years (*n = 29*)	23.7 ± 3.4	23.3 ± 3.6	22.9 ± 3.3	<0.0001	
FTI					0.23
Age <65 years	7.75 ± 3.33	7.38 ± 3.19	7.37 ± 3.32	0.0050	
Age ≥65 years	7.42 ± 2.57	7.19 ± 2.55	6.89 ± 2.31	0.0003	
SMI					0.57
Age <65 years	8.11 ± 1.38	7.97 ± 1.24	7.95 ± 1.29	0.034	
Age ≥65 years	6.98 ± 1.11	6.88 ± 1.10	6.77 ± 1.15	0.0012	
ECW/TBW ratio					0.89
Age <65 years	0.384 ± 0.007	0.383 ± 0.006	0.382 ± 0.006	0.049	
Age ≥65 years	0.397 ± 0.012	0.396 ± 0.012	0.395 ± 0.010	0.019	
HGS					0.74
Age <65 years	39.9 ± 10.3	40.8 ± 9.9	41.2 ± 10.6	0.23	
Age ≥65 years	26.5 ± 10.1	27.8 ± 10.7	27.3 ± 10.0	0.059	

### Body composition and handgrip strength changes after dapagliflozin administration in patients with or without type 2 diabetes

Changes in BMI, FTI, and SMI during the 24-week dapagliflozin therapy did not differ between patients with and without type 2 diabetes (Table 2). However, there seemed to be interactions between diabetes and non-diabetes groups in terms of changes in ECW/TBW ratio (*P = *0.070) and HGS (*P = *0.12). The ECW/TBW ratio did not change in patients without type 2 diabetes (*P = *0.37). In contrast, it decreased from 0.395 ± 0.014 at baseline to 0.393 ± 0.014 at 12 weeks (*P = *0.14) and 0.391 ± 0.012 at 24 weeks (*P = *0.004) in type 2 diabetes patients. Conversely, HGS did not change in patients with non-diabetes (*P = *0.58), whereas it tended to increase from 30.3 ± 11.8 kg at baseline to 32.0 ± 12.1 kg at 12 weeks (*P = *0.030) and 32.2 ± 12.9 kg at 24 weeks (*P = *0.087) in type 2 diabetes patients.

### Subgroup analyses stratified with age (<65 vs ≥65 years) or baseline prescription of RAS inhibitors and statins

During dapagliflozin therapy, no interaction was observed in the age-stratified groups in terms of changes in body composition and HGS (Table 2). Similarly, there was no interaction for changes in these parameters in the baseline use of RAS inhibitors and statins (Table 3).

**Table 3:** Changes in BMI, bio-impedance analysis-estimated body composition, and HGS after administration of dapagliflozin in patients with CKD, stratified by taking RAS inhibitors and statin or not at baseline.

Variables	Baseline	At 12 weeks	At 24 weeks	*P*-value	*P*-value for interaction
*RAS inhibitors*					
BMI					0.24
No (*n = 22*)	24.0 ± 4.3	23.6 ± 4.0	23.2 ± 4.1	0.0023	
Yes (*n = 33*)	26.0 ± 4.6	25.4 ± 4.4	25.2 ± 4.6	<0.0001	
FTI					0.24
No	7.34 ± 2.84	7.15 ± 2.81	6.82 ± 2.47	0.0067	
Yes	7.74 ± 3.03	7.37 ± 2.91	7.31 ± 3.04	0.0002	
SMI					0.80
No	7.15 ± 1.31	7.07 ± 1.26	7.00 ± 1.36	0.027	
Yes	7.75 ± 1.36	7.62 ± 1.26	7.54 ± 1.31	0.0024	
ECW/TBW ratio					0.93
No	0.392 ± 0.013	0.391 ± 0.012	0.390 ± 0.011	0.064	
Yes	0.390 ± 0.011	0.389 ± 0.011	0.388 ± 0.010	0.023	
HGS					0.23
No	29.2 ± 11.2	30.7 ± 11.6	29.6 ± 12.2	0.086	
Yes	35.3 ± 12.4	36.1 ± 12.2	36.8 ± 11.8	0.098	
*Statins*					
BMI					0.87
No (*n = 27*)	26.4 ± 3.9	25.8 ± 3.7	25.6 ± 3.7	0.0001	
Yes (*n = 28*)	24.1 ± 4.8	23.7 ± 4.6	23.3 ± 4.9	<0.0001	
FTI					0.87
No	8.06 ± 2.69	7.73 ± 2.65	7.58 ± 2.51	0.0020	
YES	7.11 ± 3.13	6.85 ± 3.00	6.67 ± 3.05	0.0005	
SMI					0.69
No	7.77 ± 1.33	7.64 ± 1.21	7.55 ± 1.26	0.0045	
Yes	7.26 ± 1.36	7.16 ± 1.31	7.10 ± 1.41	0.0085	
ECW/TBW ratio					0.90
No	0.390 ± 0.012	0.389 ± 0.012	0.388 ± 0.011	0.065	
Yes	0.392 ± 0.012	0.390 ± 0.011	0.389 ± 0.010	0.032	
HGS					0.72
No	35.2 ± 13.0	36.3 ± 13.0	35.8 ± 13.4	0.17	
Yes	30.6 ± 11.2	31.7 ± 11.1	32.1 ± 11.2	0.049	

### Changes in sarcopenia prevalence

**Figure 1:**
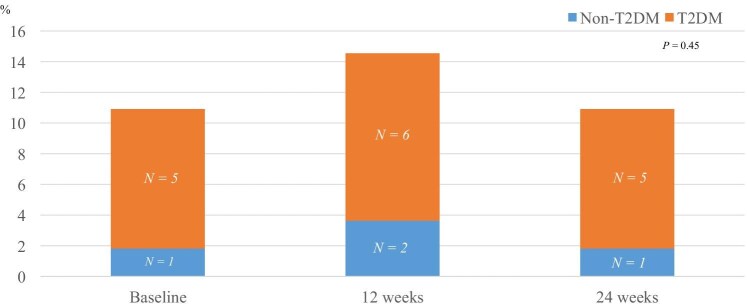
Prevalence of sarcopenia.

The sarcopenia prevalence did not change [10.9% (*n* = 6) at baseline, 14.5% (*n* = 8) at 12 weeks, and 10.9% (*n* = 6) at 24 weeks] in all the patients (*P = *0.45) (Fig. 1). The sarcopenia prevalence did not change in patients without type 2 diabetes [3.6% (*n* = 1) at baseline, 7.1% (*n* = 2) at 12 weeks, and 3.6% (*n* = 1) at 24 weeks, *P = *0.37] and in patients with type 2 diabetes [18.5% (*n* = 5) at baseline, 22.2% (*n* = 6) at 12 weeks, and 18.5% (*n* = 5) at 24 weeks, *P = *0.78].

### Associations among percentage changes during 24-week dapagliflozin therapy in BMI, FTI, SMI, and ECW/TBW ratio

The changes in BMI, FTI, SMI, and ECW/TBW ratio during 24-week dapagliflozin therapy were −3.1%, −5.8%, −2.5%, and −0.5%, respectively. The percentage changes in these variables, except the ECW/TBW ratio, were comparable in CKD patients with and without type 2 diabetes. The changes in ECW/TBW ratio (ΔECW/TBW ratio) significantly decreased more in patients with type 2 diabetes (−0.8%) than in those without type 2 diabetes (−0.2%) (*P = *0.034).

**Figure 2:**
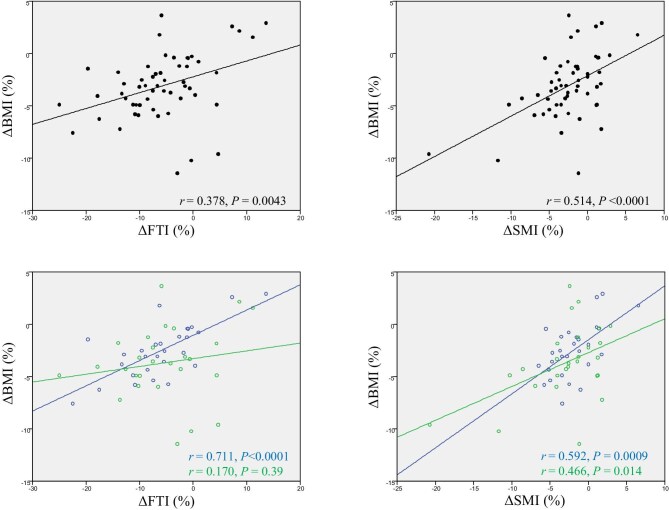
Associations between percentage changes in BMI and those in bio-impedance analysis-estimated body composition. Black lines represent longitudinal changes in all patients; green and blue lines represent longitudinal changes in patients with type 2 diabetes and without type 2 diabetes, respectively.

**Figure 3:**
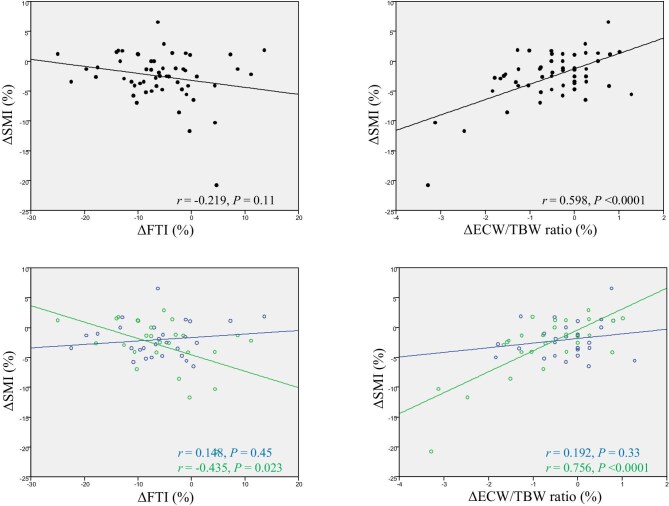
Associations between percentage changes in SMI and those in FTI and ECW/TBW ratio. Black lines represent longitudinal changes in all patients; green and blue lines represent longitudinal changes in patients with type 2 diabetes and without type 2 diabetes, respectively.

**Figure 4:**
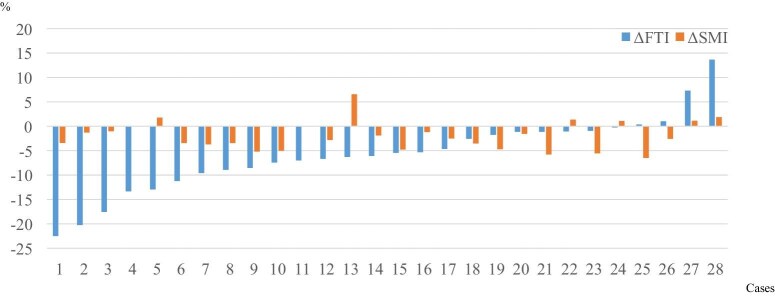
Association between ΔSMI and ΔFTI in each patient without diabetes (*n *= 28). ΔSMI and ΔFTI are shown sorted in ascending order of ΔFTI.

The percentage change in BMI (ΔBMI) was significantly correlated with those in FTI (ΔFTI) and SMI (ΔSMI) in overall patients (Fig. 2). ΔBMI was significantly correlated with ΔSMI in patients with type 2 diabetes and those without diabetes; however, ΔBMI was significantly correlated with ΔFTI only in patients with non-diabetes (Fig. 2). However, ΔSMI was significantly and negatively correlated with ΔFTI only in patients with diabetes (Fig. 3). The association between ΔSMI and ΔFTI in each patient without diabetes is shown in Fig. 4 (*n *= 28). Moreover, ΔSMI was correlated considerably with the ΔECW/TBW ratio only in patients with type 2 diabetes (Fig. 3).

### Changes in eGFR, UPCR, UACR, HbA1c, and GA

**Figure 5:**
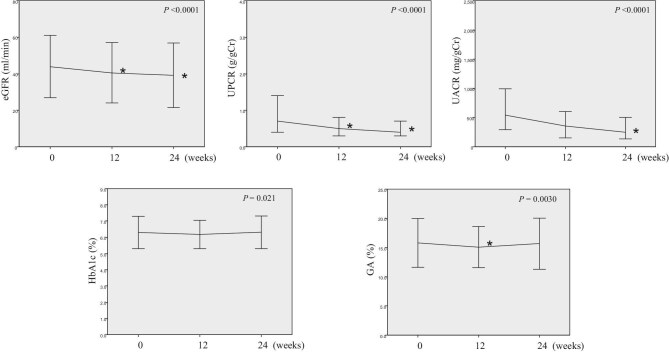
Longitudinal changes in eGFR, proteinuria, and glycemic control parameters in overall patients with CKD. *P < 0.05.

During the 24-week dapagliflozin therapy, eGFR, UPCR, UACR, HbA1c, and GA significantly changed (Fig. 5). The eGFR and UPCR at 12 and 24 weeks were significantly lower than those at baseline. UACR also tended to decrease during the follow-up period. As for glycemic control markers, HbA1c tended to decline, and GA was significantly reduced at 12 weeks; however, these markers increased back to baseline levels at 24 weeks.

### Adverse events

Diabetic ketoacidosis, severe hypoglycemia, and AKI requiring hospitalization were not observed. Only a female patient experienced cystitis and was treated with antibiotics; however, she continued to take dapagliflozin during the follow-up period.

## DISCUSSION

During the 24-week dapagliflozin therapy, there was a significant decrease in FTI, SMI, and ECW/TBW ratio with unchanged HGS in CKD patients. Additionally, sarcopenia prevalence did not change during follow-up. In the subgroup analysis, when patients were divided into type 2 diabetes and non-diabetes groups, there were no interactions in the changes in FTI and SMI. Although the ECW/TBW ratio and HGS did not change in patients without diabetes, in patients with type 2 diabetes the ECW/TBW ratio significantly decreased, and HGS tended to increase during the follow-up period. Moreover, the 24-week changes in SMI positively and significantly correlated with those in ECW/TBW ratio in CKD patients with type 2 diabetes alone. Therefore, 24-week dapagliflozin treatment might not increase sarcopenia risk in patients with CKD. Additionally, in CKD patients with type 2 diabetes, fluid volume depletion may be misdiagnosed as a decrease in skeletal muscle mass volume. However, further large-scale studies with control groups are required to validate our findings.

SGLT2 inhibitors increase urinary glucose excretion by inhibiting proximal tubular glucose reabsorption, which decreases body weight [13, 14]. SGLT2 inhibitors improve adipocyte dysfunction by decreasing leptin, increasing adiponectin, and effectively promoting lipolysis [5]. SGLT2 inhibitors decrease insulin and elevate glucagon levels, leading to limited muscle and liver absorption of amino acids and glucose, promoting hepatic gluconeogenesis and glycogenolysis, and accelerating protein breakdown in the muscle [6]. Recent meta-analyses of randomized controlled trials revealed that SGLT2 inhibitors significantly reduced body weight, BMI, and fat mass with a positive effect but also adversely reduced muscle mass compared with placebo or conventional hypoglycemic therapy in type 2 diabetes patients [3, 4]. Body weight reduction by SGLT2 inhibitors is primarily from the reduction of fat mass; the reduction of fat-free mass was attributed to ∼35% of body weight reduction in the more extended follow-up studies [15]. However, dapagliflozin administration (10 mg/day) for 12 weeks in a randomized control trial significantly decreased ∼3 kg total body weight, of which >90% was attributed to the reduction from lean body mass in patients with type 2 diabetes [16]. Thus, the effect of SGLT2 inhibitors on muscle mass is not conclusive; however, SGLT2 inhibitors might promote sarcopenia in patients with type 2 diabetes. As hypothesized, we found that 24-week dapagliflozin therapy decreased fat and muscle mass in CKD patients. A similar decrease in fat and muscle mass was observed in patients with CKD with and without type 2 diabetes. Thus, special attention should be paid to the adverse effects of dapagliflozin in reducing skeletal muscle mass.

In our study, HGS did not decrease, and it even increased in CKD patients. HGS did not change in patients without diabetes but tended to increase in patients with type 2 diabetes. Because previous studies did not evaluate muscle quality, HGS measurements and/or physical activities, diagnosing sarcopenia was challenging. Recently, an experimental study with *db*/*db* mice (fatty type 2 diabetes model) demonstrated that administering luseogliflozin significantly increased grip strength [17]. The study proposed the importance of intramuscular fatty acid metabolism and gene expression influenced by the extracellular lipidome modified by SGLT2 inhibitors as a mechanism for SGLT2 inhibitors alleviating sarcopenia [17]. A sub-analysis of a randomized control trial comparing two groups of type 2 diabetes receiving sitagliptin adding ipragliflozin or metformin showed that HGS slightly increased at 24 weeks; however, these changes were insignificant between groups [18]. SGLT2 inhibitors, including ipragliflozin, luseogliflozin, and dapagliflozin, reportedly increased HGS during ∼10 weeks of observational periods; however, the precise mechanisms remain unknown [19]. Our findings were consistent with these results as we found that HGS tended to increase in CKD patients with type 2 diabetes. Moreover, sarcopenia prevalence did not increase with dapagliflozin therapy in overall patients, and even in the subgroups divided into type 2 diabetes and non-diabetes. Hence, short-term dapagliflozin therapy for CKD may likely be safe against sarcopenia. However, more long-term observational periods are required to confirm dapagliflozin safety in patients with CKD.

Sarcopenia is prevalent in older patients; therefore, dapagliflozin can differently affect body composition and HGS according to age. However, during dapagliflozin therapy there were similar effects on these parameters in age-stratified groups (<65 vs ≥65 years) in the present study. In addition, medications can also affect body composition and HGS. Some observational studies have reported that the use of RAS inhibitors seems to positively affect sarcopenia in patients with heart failure and those on hemodialysis [20, 21]. Conversely, loop diuretics, via a decrease in myoblast fusion, and statins, via mitochondrial dysfunction, were theoretically considered as medications for drug-related sarcopenia [22]. In a clinical setting, an observational study reported that loop diuretics were associated with a risk of sarcopenia in patients with CKD [23], whereas a cross-sectional study reported that statins were associated with a reduced likelihood of sarcopenia in patients with heart failure [24]. Additionally, in animal studies, GLP-1RA may have protective effects on skeletal muscle against sarcopenia by increasing insulin sensitivity, inhibiting myostatin expression, and increasing mitochondrial biogenesis [22]. However, some clinical studies reported that GLP-1RA decreased lean body mass, ranging between 20% and 50% of total weight loss, possibly mediated by a decrease in appetite or a direct effect on muscle [25]. In the present study, no interaction was observed between changes in body composition and HGS in the baseline use of RAS inhibitors and statins during dapagliflozin therapy. Conversely, we believe that diuretics and GLP-1RA might be potential confounders; however, further large-scale studies are required to determine how these medications affect dapagliflozin administration and changes in body composition and HGS.

Additional analyses revealed that there was a significant decrease in the ECW/TBW ratio during 24-week dapagliflozin administration in all patients. Only a few reports have investigated the association between the ECW/TBW ratio and clinical outcomes. A cross-sectional study reported that the ECW/TBW ratio was independently associated with coronary artery calcification, a marker of cardiovascular disease, in patients with pre-dialysis CKD (per 0.01 increase of ECW/TBW, odds ratio 1.17) [26]. In addition, an observational study reported that a higher ECW/TBW ratio (0.39–0.40 and 0.40) was independently associated with CKD progression (adjusted hazard ratio: 1.45 and 1.78, respectively) compared with the normal ECW/TBW ratio (<0.38) [27]. Moreover, an increase in the ECW/TBW ratio was also independently associated with CKD progression (adjusted hazard ratio: 1.40) compared with no change or decrease in the ECW/TBW ratio. In the present study, the ECW/TBW ratio was considerably higher in patients with type 2 diabetes than in those without diabetes at baseline. It significantly decreased from 0.395 to 0.391 during the follow-up only in patients with type 2 diabetes. Moreover, ΔECW/TBW ratio during follow-up positively and significantly correlated with ΔSMI in CKD patients with type 2 diabetes alone. Recently, a sub-analysis of the EMPA-KIDNEY trial revealed that empagliflozin significantly reduced fluid overload compared with placebo in the CKD population with or without diabetes [28]. Therefore, weight loss from SGLT2 inhibitors may be due to, in part, a loss of excess body water based on osmotic diuresis and natriuresis. Hence, our results suggest that skeletal muscle mass volume might be overestimated at baseline, and dapagliflozin therapy affects decreasing excess fluid overload in CKD patients with type 2 diabetes. In CKD patients, bio-impedance analysis-estimated SMI is affected by the patient's fluid status; i.e. when body weight significantly decreased from 72.9 ± 17.8 to 70.9 ± 19.9 kg after a hemodialysis session, multi-frequency bio-impedance analysis-estimated fat-free mass also significantly decreased from 51.8 ± 19.2 to 48.1 ± 11.8 kg in patients on hemodialysis [29]. Therefore, future studies must assess muscle mass volume using methods less affected by fluid volume, such as CT [7, 30].

By contrast, in patients without diabetes, ΔBMI was significantly correlated with ΔSMI and ΔFTI; however, there was no correlation between ΔSMI and ΔFTI. FTI was more likely to decrease compared with SMI; however, some patients had ΔSMI much larger than ΔFTI, and others had ΔFTI much larger than ΔSMI. The reason remains unclear; there seemed to be two different populations of patients without type 2 diabetes: some are likely to decrease SMI, and others are likely to decrease FTI by administration of dapagliflozin.

In the present study, patients were similar in age, predominantly male, had lower BMI, similar eGFR, lower UACR, and proportions taking lower RAS inhibitors and diuretics and similar statins compared with the dapagliflozin treatment arm in the DAPA-CKD trial [9]. The low proportion of treatment with RAS inhibitors may be due to the reflection of clinical practice in Japan [31]. In this study, changes in eGFR and UACR were similar to those in the DAPA-CKD trial [32, 33]. Severe adverse effects such as diabetic ketoacidosis, severe hypoglycemia or hyperglycemia, hyperkalemia, severe dehydration, acute kidney injury, bone fracture, and Fournier's gangrene were not observed in this study. A female patient developed cystitis; however, she continued to receive dapagliflozin after antibiotic therapy.

The study had several limitations. First, our findings may not be generalizable to all populations with CKD as this single-arm, prospective, observational study included a small number of Japanese patients with CKD. In addition, our study cohort was almost entirely male; therefore, this might have affected the measurements of body composition, considering the fact that the effects on body composition could differ by sex. Second, because the efficacy of dapagliflozin has already been established in patients with CKD, establishing a group without dapagliflozin treatment was ethically impossible. Consequently, we performed a sub-study in which the patients were divided into groups with and without type 2 diabetes. Third, the accomplishment of diet and exercise therapy and other lifestyle modifications, which could affect body composition and muscle strength independently of dapagliflozin, was difficult to verify in this study. Exercise therapy was not monitored or instructed, and only regular walking was recommended in the present study. Organized exercises, including resistance training, can be proposed to conserve skeletal muscle volume and improve physical function in patients using SGLT2 inhibitors [34, 35]. However, in this study the extent of spontaneous exercise or training in each patient might have affected the body composition or HGS results. Fourth, the follow-up period was relatively short (only 24 weeks) to monitor sarcopenia prevalence and determine the safety of dapagliflozin. Therefore, further longitudinal randomized clinical trials, including other ethnic groups with long-term follow-up periods, considering appropriate exercise therapy, are required to provide clear evidence of the safety of dapagliflozin in terms of sarcopenia.

In conclusion, during short-term administration of dapagliflozin, there was a decrease in FTI, SMI, and ECW/TBW ratio with unchanged HGS; the prevalence of sarcopenia did not change in CKD patients. Additionally, subgroup analyses showed that the ECW/TBW ratio significantly decreased, and HGS tended to increase only in patients with type 2 diabetes. Additionally, changes in SMI during follow-up were significantly correlated with those in the ECW/TBW ratio in patients with CKD with type 2 diabetes alone. Therefore, short-term administration of dapagliflozin might be safe for sarcopenia in patients with CKD; however, changes in body composition and HGS by dapagliflozin might differ between patients with and without type 2 diabetes.

## Data Availability

The data underlying this article will be shared on reasonable request to the corresponding author.
